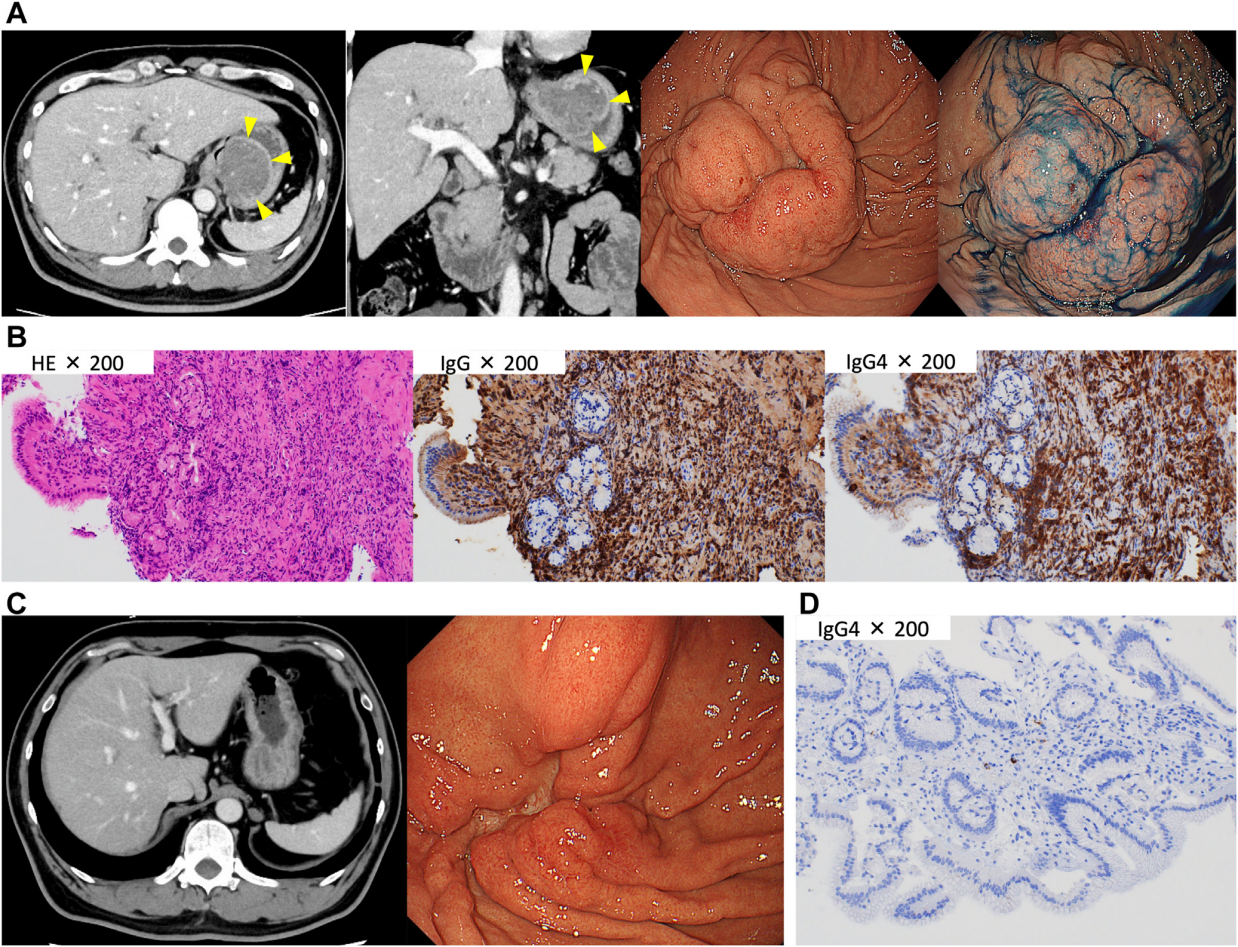# A Rare Cause of Submucosal Tumor-like Lesion

**DOI:** 10.1016/j.gastha.2025.100662

**Published:** 2025-03-22

**Authors:** Tomoya Nakamura, Yujiro Kawakami, Hiroshi Nakase

**Affiliations:** Department of Gastroenterology and Hepatology, Sapporo Medical University School of Medicine, Sapporo, Japan

A 49-year-old man presented with upper abdominal pain. Laboratory tests revealed normal serum carcinoembryonic antigen and carbohydrate antigen 19-9 levels and elevated serum immunoglobulin G4 (IgG4) levels (1412 mg/dL). Contrast-enhanced computed tomography revealed bilateral submandibular gland enlargement, diffuse pancreatic enlargement, and a 55-mm enhanced mass in the stomach fundus (Figure A). Esophagogastroduodenoscopy revealed a submucosal tumor (SMT)–like lesion with an ulcer in the fundus (Figure A). We performed endoscopic biopsy of the ulcer part of the SMT-like lesion. Histopathology revealed >10 IgG4-positive lymphoplasmacytic cells/high-power field, with an IgG4/immunoglobulin-positive cell ratio of >40% (Figure B). Based on the laboratory, radiological, and pathological findings, the patient was diagnosed with IgG4-related gastrointestinal diseases (IgG4-GIDs). Treatment was initiated with 45 mg of prednisolone daily, with the dosage was tapered gradually. Contrast-enhanced computed tomography and esophagogastroduodenoscopy performed 3 months later revealed drastic shrinkage of the gastric lesion (Figure C). No IgG4-positive plasma cell infiltration was detected in endoscopic biopsy specimens of the lesion (Figure D).

IgG4-GID is associated with morphological changes, including ulcers, strictures, and SMT. Gastrointestinal lesions associated with IgG4-related disease are often misdiagnosed as gastrointestinal stromal tumors based on imaging findings. IgG4-GID is commonly diagnosed postoperatively, with few cases diagnosed through endoscopic biopsy.

**Figure:**